# Gene Cascade Shift and Pathway Enrichment in Rat Kidney Induced by Acarbose Through Comparative Analysis

**DOI:** 10.3389/fbioe.2021.659700

**Published:** 2021-05-21

**Authors:** Chun-Yue Weng, Mo-Han Zhu, Ke-Lei Dai, Zhe-Yan Mi, Yuan-Shan Wang, Zhi-Qiang Liu, Yu-Guo Zheng

**Affiliations:** ^1^The National and Local Joint Engineering Research Center for Biomanufacturing of Chiral Chemicals, Zhejiang University of Technology, Hangzhou, China; ^2^Key Laboratory of Bioorganic Synthesis of Zhejiang Province, College of Biotechnology and Bioengineering, Zhejiang University of Technology, Hangzhou, China; ^3^Engineering Research Center of Bioconversion and Biopurification, Ministry of Education, Zhejiang University of Technology, Hangzhou, China

**Keywords:** acarbose, *Rattus norvegicus*, kidney, comparative analysis, lipid metabolism

## Abstract

Acarbose is an effective anti-diabetic drug to treat type 2 diabetes mellitus (T2DM), a chronic degenerative metabolic disease caused by insulin resistance. The beneficial effects of acarbose on blood sugar control in T2DM patients have been confirmed by many studies. However, the effect of acarbose on patient kidney has yet to be fully elucidated. In this study, we report in detail the gene expression cascade shift, pathway and module enrichment, and interrelation network in acarbose-treated *Rattus norvegicus* kidneys based on the in-depth analysis of the GSE59913 microarray dataset. The significantly differentially expressed genes (DEGs) in the kidneys of acarbose-treated rats were initially screened out by comparative analysis. The enriched pathways for Gene Ontology (GO) and Kyoto Encyclopedia of Genes and Genomes (KEGG) analyses were further identified. The protein-protein interaction (PPI) analysis for DEGs was achieved through the STRING database mining. Pathway interrelation and hub genes for enriched pathways were further examined to uncover key biological effects of acarbose. Results revealed 44 significantly up-regulated genes and 86 significantly down-regulated genes (130 significant differential genes in total) in acarbose-treated rat kidneys. Lipid metabolism pathways were considerably improved by acarbose, and the physical conditions in chronic kidney disease (CKD) patients were improved possibly through the increase of the level of high-density lipoprotein (HDL) by lecithin-cholesterol acyl-transferase (LCAT). These findings suggested that acarbose may serve as an ideal drug for CKD patients, since it not only protects the kidney, but also may relieve the complications caused by CKD.

## Highlights

– Comparative analysis of acarbose-treated rat kidney samples were performed.
– Totally 130 significant differential genes were identified.
– Acarbose can markedly affect lipid metabolism and electrolytes in rat kindey.
– The findings provide targets for molecular mechanism researches.
– Acarbose could be ideal drug candidate for chronic kidney disease patients.

## Introduction

Diabetes mellitus is a chronic degenerative metabolic disease caused by insulin resistance and islet β-cell dysfunction (Wu et al., 2014). In 2019, approximately 463 million adults (20–79 years) were living with diabetes (Saeedi et al., 2019). Over 90% of all diabetes mellitus are type 2 diabetes mellitus (T2DM). Insufficient insulin production from beta cells during insulin resistance is considered to be the cause of T2DM (Ahrén, 2005).

Acarbose is an anti-diabetic drug used to treat T2DM, which is an oligosaccharide that reversibly inhibits intestinal α-glucosidase enzymes and is responsible for digesting complex carbohydrates and disaccharides to absorb monosaccharides (Hillebrand et al., 1979; Ibrahim et al., 2018). Acarbose has competitive inhibitory activities toward intestinal maltase, sucrase, dextrinase and glycoside hydrolases, including pancreatic alpha-amylase and alpha-glucosidase in the brush border of the small intestines, and thereby delays carbohydrate absorption and controls postprandial hyperglycemia (Wehmeier and Piepersberg, 2004). The digestion rate of complex carbohydrates reduces as these enzyme systems have been inhibited. Since the carbohydrates are not broken down into glucose, less glucose is absorbed (Scheen, 1998). However, since T2DM becomes more prevalent worldwide, the demand for acarbose increases rapidly. Acarbose is produced in large-scale fermentation by *Actinoplanes* sp. SE50/110, which was obtained from rounds of mutagenesis and selection. Recently, comparative genome, transcriptome, and proteome analyses have provided system-level understanding of *Actinoplanes* sp. SE50/110 and insights into the mechanisms of acarbose overproduction (Wendler et al., 2016; Xie et al., 2019). A highly efficient genetic manipulation system was established for *Actinoplanes* sp. SE50/110 and was used to delete *melC2* (a tyrosinase gene) and *treY* (a maltooligosyltrehalose synthase gene), which eliminated the formation of eumelanin and the by-product component C, resulting in a 35% increase of acarbose titer (Gren et al., 2016; Zhao et al., 2017). Additionally, in the latest research, strategies were implemented by minimizing the flux to the shunt products and maximizing the supply of the amino-deoxyhexose moiety, which increased the acarbose titer to 7.4 g/L (Zhao et al., 2020). Moreover, the effect of different saccharides on acarbose production by *Actinoplanes* has been studied and the transcriptome analysis revealed molecular signature of saccharide impact on acarbose biosynthesis (Weng et al., 2020, 2021). Acarbose also showed positive effects on diabetes-associated hyperlipidemia and decreased various blood lipid levels (Kado et al., 1998; Ji, 2017). Furthermore, the benefits in lipid profile associated with acarbose therapy were considered to be independent of glycemic control (Kawamura et al., 1998).

The beneficial effects of acarbose on diabetes have been confirmed by many studies (Hanefeld, 2007; Wei and Xu, 2019). However, few studies reported the global genetic effect of acarbose on kidneys, which suffers a lot from the side effects of various drugs. In this study, systematic bioinformatics analyses for a microarray dataset, GSE59913, have been performed to identify key differential genes, pathways, modules, and protein-protein interactions (PPIs) that may be critical for changes in multiple physiological activities in the kidney of rats treated with acarbose. This study uncovered 130 significant differential genes in acarbose-treated rat kidney samples. Lipid metabolism pathways were considerably enriched. Results suggested that acarbose may be an ideal drug candidate for chronic kidney disease (CKD) patients, because it can not only protect the kidney, but also relieve the complications caused by CKD.

## Materials and Methods

### Microarray Data

The microarray dataset GSE59913 was downloaded from GEO website^1^. GSE59913 was composed of transcriptional profiling data using the GE Healthcare/Amersham Biosciences CodeLink^TM^ UniSet Rat I Bioarray from *Rattus norvegicus* kidney samples. Two groups were selected that have 3 samples of each treated with either 2000 mg/kg acarbose (referred as group Acarbose, including GSM1454696, GSM1454704, and GSM1454725 samples) or water (referred as group Water, including GSM1455062, GSM1455063, and GSM1455064 samples) on Day 3 for our analysis.

### Differentially Expressed Gene (DEG) Identification, Principal Component Analysis (PCA) and Heat Map Construction

The Series Matrix File of GSE59913 was downloaded and analyzed with customized R code to identify DEGs, draw heat maps and perform PCA analysis (Roweis and Saul, 2000; Price et al., 2006; Trapnell et al., 2012). In the current study, genes with logFC ≥ 1.5 or logFC ≤ −1.5 and *p* value < 0.05 were regarded as DEGs, while Water group served as controls. Gene expressions of GSE59913 for different samples were utilized for PCA. The top 300 significantly changed genes (including 150 up-regulated and 150 down-regulated genes) were utilized for the presentation of heat map.

### Gene Ontology (GO) and Kyoto Encyclopedia of Genes and Genomes (KEGG) Pathway Enrichment Analyses of DEGs

The GO term enrichment analyses were used to explore the biological significance of DEGs according to gene ontologies, including their functions in biological process, cellular component, and molecular function (Ashburner et al., 2000; Attrill et al., 2019). The gene names of DEGs were input to the Gene Ontology project^2^ to export the enriched terms (Attrill et al., 2019). The KEGG pathways were examined to reveal the pathway enrichment of DEGs between different groups so that DEGs can be condensed into enriched pathways^3^ (Kanehisa and Sato, 2020). *P* < 0.05 was considered statistically significant (Fu et al., 2012).

### Protein-Protein Interaction (PPI) Network Building, Interrelation Analyses Between Pathways and Hub Gene Analyses

The PPI information was evaluated by the STRING database version 11.0^4^ to reveal the interactions between various protein families (Szklarczyk et al., 2015). Network of DEGs was conducted using settings including experiments, text mining, database, coexpression, neighborhood, gene-fusion, and cooccurrence box checked. MCODE (Version 1.5.1), a plugin of Cytoscape (Version 3.7.1), was used to filter modules with significant roles from the PPI network (Bader and Hogue, 2003; Saito et al., 2012). Modules with MCODE score ≥ 3 and node number > 3 were presented as significant modules. ClueGo plugin (Version 2.5.4) was used to perform the interrelation analyses between pathways, with biological process, cellular component, molecular function, and KEGG terms/pathways selected (Attrill et al., 2019). Only terms/pathways with *p* < 0.05 were presented. CytoHubba plugin (Version 0.1) was used to analyze the hub genes for enriched pathways with 12 algorithms examined so that key genes in the differential genes can be revealed (Chin et al., 2014).

## Results

### Identification of Significantly Differentially Expressed Genes (DEGs)

**FIGURE 1:**
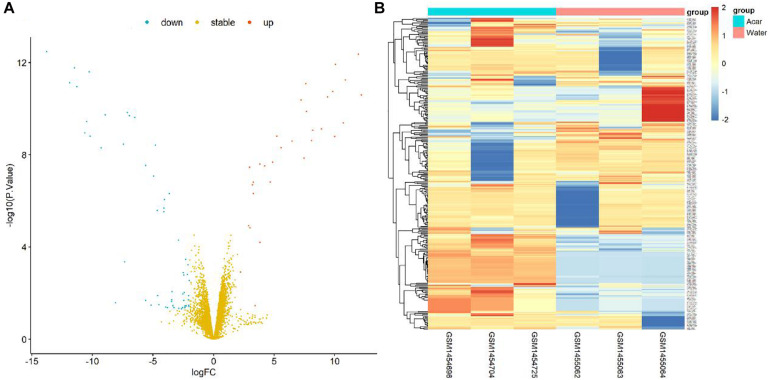
Volcano plot and heatmap of differential genes between Acarbose and Water. **(A)** All 6084 differential genes were utilized for volcano plot. Red: significantly up-regulated in Acarbose; blue: significantly down-regulated in Acarbose; yellow: no significant change. **(B)** The top 150 most significantly up-regulated and 150 most significantly down-regulated genes were utilized for heatmap presentation. Red, up-regulated genes in Acarbose; blue, down-regulated genes in Acarbose. Samples showed distinct gene expression patterns between the two groups, but comparable expression patterns within a group.

The Principal Components Analyses (PCA) revealed that Acarbose and Water samples were significantly different from each other. The first two principal components accounted for 61.8% of the data variance (Figure 1).

Furthermore, the DEG pattern was analyzed. The expressions of 6084 identified genes were either up-regulated or down-regulated between Acarbose and Water. Totally, 44 up-regulated genes and 86 down-regulated genes (130 in total) were recognized as significantly changed DEGs based on our criteria. All 6084 genes were plotted in the volcano plot, as shown in Figure 1A. The up-regulated genes were represented in red, the down-regulated genes were in blue, and the stable genes whose expressions showed no significant change were in yellow.

The top 300 DEGs (150 up-regulated and 150 down-regulated) were showed in the heatmap, which were well clustered between Acarbose and Water (Figure 1B). Based on these top DEGs, Acarbose and Water samples demonstrated a significantly distinct pattern between groups, but a similar expression pattern within a group (Figure 1B). Therefore, the top DEGs can be employed to differentiate between Acarbose and Water samples.

### Gene Ontology (GO) Term Enrichment Analyses and Kyoto Encyclopedia of Genes and Genomes (KEGG) Pathway Analyses of DEGs

Search Tool for the Retrieval of Interacting Genes/Proteins (STRING) database was used to search the names of all the significantly altered genes. For the cellular component domain, DEGs were significantly enriched in the chylomicron, high-density lipoprotein particle, triglyceride-rich plasma lipoprotein particle, very-low-density lipoprotein particle, lipoprotein particle, plasma lipoprotein particle, and protein-lipid complex (Table 1).

**TABLE 1:** Enriched GO pathways in Acarbose compared to Water.

Term ID	Term description	Observed gene count	Over/Under	Fold enrichment	False discovery rate
**Cellular component**					
GO:0042627	Chylomicron	3	+	57.39	2.91 × 10^–5^
GO:0034364	High-density lipoprotein particle	5	+	50.11	3.96 × 10^–5^
GO:0034385	Triglyceride-rich plasma lipoprotein particle	4	+	46.76	8.61 × 10^–5^
GO:0034361	Very-low-density lipoprotein particle	4	+	46.76	1.82 × 10^–4^
GO:1990777	Lipoprotein particle	5	+	37.58	2.05 × 10^–4^
**Molecular function**					
GO:0003870	5-aminolevulinate synthase activity	2	+	>100	1.37 × 10^–6^
GO:0016749	N-succinyltransferase activity	2	+	>100	3.12 × 10^–6^
GO:0004937	Alpha1-adrenergic receptor activity	2	+	>100	1.27 × 10^–4^
GO:0016748	Succinyltransferase activity	2	+	>100	2.63 × 10^–4^
GO:0055102	Lipase inhibitor activity	3	+	48.56	3.28 × 10^–4^
**Biological process**					
GO:0001983	Baroreceptor response to increased systemic arterial blood pressure	2	+	> 100	1.08 × 10^–11^
GO:0061368	Behavioral response to formalin induced pain	2	+	> 100	3.08 × 10^–11^
GO:0061366	Behavioral response to chemical pain	2	+	> 100	4.63 × 10^–11^
GO:0001994	Norepinephrine-epinephrine vasoconstriction involved in regulation of systemic arterial blood pressure	2	+	> 100	2.22 × 10^–10^
GO:0010901	Regulation of very-low-density lipoprotein particle remodeling	2	+	> 100	2.86 × 10^–10^

*Top 5 most significantly enriched items were shown. “+” in the “Over/Under” column indicates overexpression in Acarbose, while “−” indicates under expression.*

For the molecular function domain, DEGs were significantly enriched in 5-aminolevulinate synthase activity, N-succinyltransferase activity, alpha1-adrenergic receptor activity, succinyltransferase activity, lipase inhibitor activity, adrenergic receptor activity, and steroid binding (Table 1).

For the biological process domain, DEGs were significantly enriched in baroreceptor response to increased systemic arterial blood pressure, behavioral response to formalin induced pain, behavioral response to chemical pain, norepinephrine-epinephrine vasoconstriction involved in the regulation of systemic arterial blood pressure, regulation of very-low-density lipoprotein particle remodeling and regulation of systemic arterial blood pressure by carotid sinus baroreceptor feedback, as shown in the biological process part of Table 1.

KEGG analysis demonstrated that DEGs were mainly involved in neuroactive ligand-receptor interaction (Supplementary Table 1).

### Construction of Protein-Protein Interaction (PPI) Network and Analyses of Interrelation Between Pathways

**FIGURE 2:**
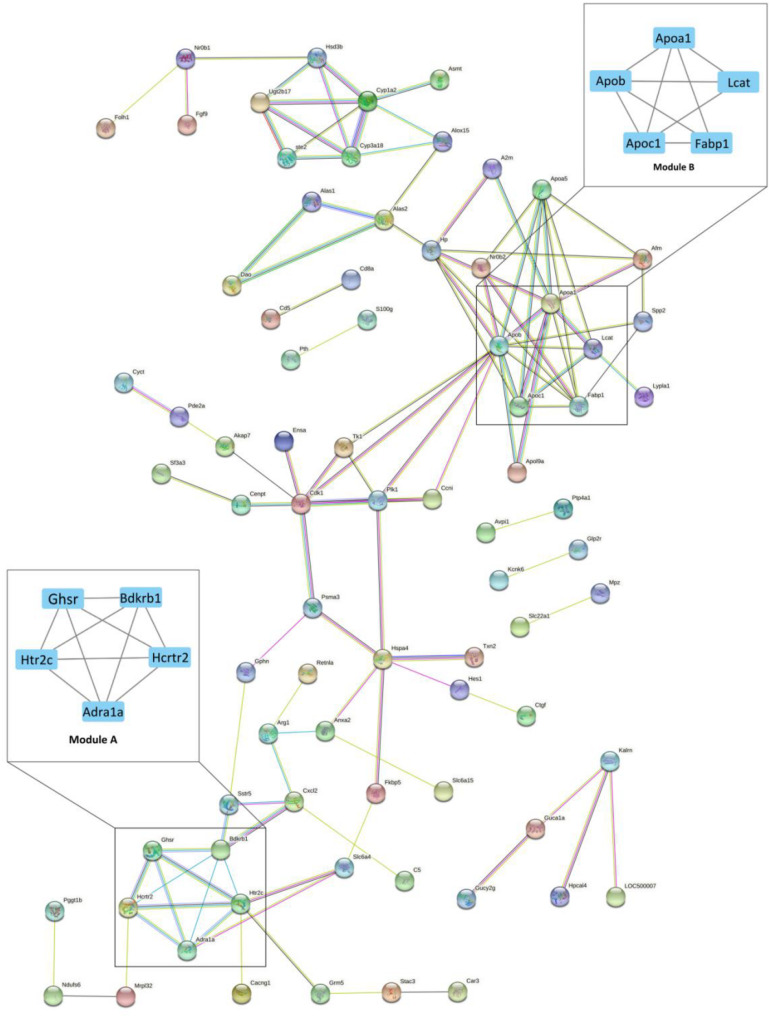
The protein-protein interaction (PPI) network of differentially expressed genes (DEGs) between Acarbose and Water. The 130 significant DGEs were employed for PPI network construction. Each node represented a protein, and each connection represented an interaction. Module A and B were the most significantly enriched functional protein modules. Module A: the hormone receptor module; Module B: the lipoprotein module. The cutoff for module filtration is: MCODE score > 3, and nodes > 3.

**FIGURE 3:**
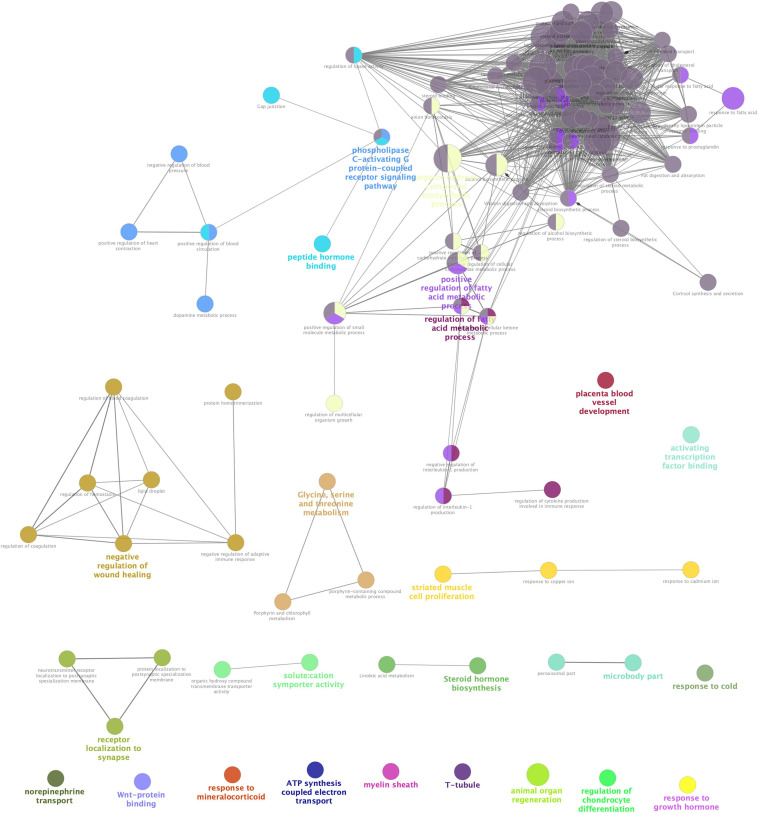
Interrelation analyses between differential pathways between Acarbose and Water. The interrelation of biological process (GO), cellular component (GO), molecular function (GO), and KEGG for the differentially expressed genes (DEGs) between Acarbose and Water was analyzed using the ClueGo plugin in Cytoscape. Each node corresponded to a term, the size of the node corresponded to the universality of the term in the enriched genes, and the connection between the nodes showed the correlations between different terms. Modules in different colors represented different functions. The selection threshold for terms/pathways is *p* < 0.05.

STRING analysis of all significant DEGs revealed 118 nodes and 114 edges (Figure 2). The modules were determined based on the PPI network. As shown in Figure 2, **5** proteins formed module A, including growth hormone secretagogue receptor (Ghsr), bradykinin receptor B1 (Bdkrb1), 5-hydroxytryptamine receptor 2C (Htr2c), hypocretin receptor 2 (Hcrtr2), and adrenoceptor alpha 1A (Adra1a), while apolipoprotein A1 (Apoa1), apolipoprotein B (Apob), apolipoprotein C1 (Apoc1), lecithin cholesterol acyltransferase (Lcat), and fatty acid binding protein 1 (Fabp1) formed module B. Correlation analysis was conducted by accessing the biological process, cellular component, molecular function, and KEGG terms/pathways through ClueGO. All the DEGs were mainly enriched in high-density lipoprotein particle, regulation of fatty acid metabolic process, organic hydroxy compound biosynthetic process, phospholipase C-activating G protein-coupled receptor signaling pathway, peptide hormone binding, negative regulation of wound healing, glycine, serine and threonine metabolism, receptor localization to synapse, striated muscle cell proliferation, steroid hormone biosynthesis, microbody part, and solute: cation symporter activity. Most of the genes were involved in two or more processes (Figure 3).

### Hub Gene Analyses

All DEGs were analyzed by the CytoHubba app (a plugin of Cytoscape) for the hub gene determination to identify the key genes that cause gene expression cascade change. Twelve algorithms were utilized for identification of the most reliable hub genes, and 36 hub genes were found. The top 10 hub gene products are as follows: Cdk1, Apob, P1k1, Hp, Hspa4, Apoc1, Psma3, Ccni, Fabp1, Apoa1, and Apoa5 (Supplementary Table 2). Most of the hub genes are related to lipoproteins (Figure 2, Module B).

## Discussion

In this study, DEGs between samples of Acarbose and Water groups were identified by comparative analysis. Analyses of PCA, GO, KEGG, PPI network, and interrelation between pathways were conducted. In total, 44 significantly up-regulated and 86 significantly down-regulated genes from GSE59913 were presented. Bioinformatics analyses were conducted based on these DEGs.

Firstly, among the pathway enrichment analyses, cell component enrichment (GO) analysis showed that the DEGs were mainly concerned about lipoproteins, which was consistent with the previous studies that acarbose may contribute to the changes in blood lipoprotein composition (Festa et al., 1999; Reddy et al., 2010; Hedrington and Davis, 2019). Up to 97% of patients with diabetes are dyslipidemic because the flux of free fatty acids increases during hyperglycemia, which also contributes to the formation of atherosclerosis (Festa et al., 1999; Reddy et al., 2010). Previous research investigated the influences of acarbose on postprandial lipid metabolism and found that the lipoprotein remnants at acute phase and acute post-prandial increase of triglycerides were ameliorated by the drug (Hedrington and Davis, 2019). According to the results of cell component enrichment analyses (Table 1), the effects of postprandial lipid metabolism are related to the regulation of lipoproteins, including chylomicron, high-density lipoprotein particle, etc., which will be discussed further below. Secondly, molecular function (GO) enrichment analyses showed that DEGs mainly influence protein binding. Lipid binding and phosphatidylcholine-sterol O-acyltransferase activator activity were also affected, showing the effect on the blood and the lipids in it, which will be discussed in more details with the networks of DEGs. Thirdly, biological process (GO) enrichment analyses showed that DEGs were mainly included in the regulation of transport, response to oxygen-containing compound and response to the endogenous stimulus. Acarbose has been shown to down-regulate oxidative stress and platelet activation and may therefore have potential cardioprotective functions (Santilli et al., 2010). The results of the analyses further supported that acarbose did have a positive effect on oxidative stress in the body.

The current study analyzed the network of DEGs and the interrelation of pathways (Figure 2). Bradykinin is a peptide that causes vasodilation and therefore reduces blood pressure (Dendorfer et al., 2001). As a hormone receptor module, Module A shows that acarbose affects blood pressure by the regulation of bradykinin receptor B1 (Bdkrb1). Additionally, 5-hydroxytryptamine receptor 2C (Htr2c) and hypocretin receptor 2 (Hcrtr2) were reported to participate in the regulation of intestinal movements and lipid metabolism, respectively (Berger et al., 2009; Skrzypski et al., 2011). Module B is the lipoprotein module, including several kinds of apolipoprotein, fatty acid binding protein and lecithin cholesterol acyltransferase. Blood lipids are mainly fatty acids and cholesterol, which can be carried in the blood stream by plasma lipoprotein particles (Shepherd et al., 1987). Acarbose has shown beneficial effects on hyperlipidemia caused by diabetes and decreases in free fatty acids and cholesterol levels (Patel, 2015), which result from the regulation of fatty acid binding protein and lecithin cholesterol acyltransferase according to Module B. Moreover, Hub Gene Analyses also showed a similar result to Module B, which further confirms the role of acarbose in blood lipids (Supplementary Table 2). Studies have shown the relationship between these two proteins and their corresponding lipids, but the mechanism of the actions between these two proteins and acarbose still remain unresolved, and further research should be performed to better explain how acarbose affects blood lipids (Yu et al., 2017). In addition, the effects related to kidney diseases also need to be noted, because renal dysfunction is related to many disorders in lipoprotein metabolism, leading to dyslipidemia and accumulation of atherosclerotic particles (Vaziri, 2014). Moreover, lecithin-cholesterol acyl-transferase (LCAT) can be observed in both Module B and hub genes, and its impact on chronic kidney disease (CKD) needs attention. LCAT deficiency is reported to contribute to diminished HDL cholesterol content and impaired HDL maturation in advanced CKD patients, which further increases oxidative stress and cardiovascular mortality in patients with CKD (Moradi et al., 2009; Ribeiro et al., 2012). However, acarbose has been shown to increase the level of HDL cholesterol, and our result suggests that this may work through effects on LCAT, and therefore may help improve physical conditions in CKD patients (Monami et al., 2012). Previous studies have shown that acarbose can protect kidney function by reducing the kidney’s filtered ammonia and nitrogen load (Evenepoel et al., 2006). Current research shows that acarbose may be an ideal drug candidate for CKD patients, because it not only protects the kidney, but also may relieve the complications caused by CKD.

Differentially expressed genes showed enrichment in lipoprotein particle, regulation of fatty acid metabolic process, peptide hormone binding and steroid hormone biosynthesis, which have been discussed before. However, other kinds of enrichment need more discussion. Solute: cation symporter activity, ATP synthesis coupled electron transport and response to mineralocorticoid related events can be seen in Figure 3, which indicates that acarbose affects a series of events about electrolytes. Acarbose may cause abnormal levels of potassium and sodium, and remain a risk of electrolyte disturbances (Khatoon and Najam, 2012). Enrichment of DEGs showed that Na^+^-K^+^-ATPase might play an important role, while mineralocorticoids also take part in this progress, because they influence electrolyte balance and fluid balance (Feraco et al., 2019). As the mineralocorticoids are produced in the adrenal cortex, some progresses about these tissues were also influenced (Table 1). Electrolyte imbalances should be considered as this may cause kidney damage, so that both ATPase and mineralocorticoids should be included into observation. Moreover, Ca^2+^, as a part of electrolyte, should also be monitored because T-tubule has been included in the DEGs enrichment, which is studded with proteins including L-type calcium channels, sodium-calcium exchangers, calcium ATPases, and Beta adrenoceptors (Hong and Shaw, 2017).

## Conclusion

In this study, comprehensive bioinformatics analyses of gene expression profiles of Acarbose samples compared with Water samples in the *R. norvegicus* kidney were performed, and key genes and pathways were identified. This study revealed that the effect of acarbose on blood sugar control was mainly through the regulation of lipid metabolism and electrolytes, which was consistent with the previous studies. This finding can reveal a series of targets for further research of molecular mechanisms. Also, new insights into acarbose as an ideal drug for CKD patients had been presented, but the possibility of kidney damage from electrolyte imbalances also needs to be treated with caution. However, this study mainly focused on bioinformatic analysis of differential genes and pathways, corroborative bench work is still necessary to confirm the proposed regulation network.

## Data Availability Statement

The datasets presented in this study can be found in online repositories. The names of the repository/repositories and accession number(s) can be found in the article/Supplementary Material.

## Author Contributions

C-YW: data analysis, validation, writing-original draft, and funding acquisition. M-HZ: investigation, methodology, and writing original draft. K-LD: manuscript revision, writing-reviewing, and editing. Z-YM: methodology and data validation. Y-SW: writing-reviewing and editing. Y-GZ: conceptualization and resources. Z-QL: resources, writing-reviewing, and editing. All authors contributed to the article and approved the submitted version.

## Conflict of Interest

The authors declare that the research was conducted in the absence of any commercial or financial relationships that could be construed as a potential conflict of interest.

## Footnotes

1 https://www.ncbi.nlm.nih.gov/geo/

2 http://geneontology.org/

3 https://www.genome.jp/kegg/

4 https://string-db.org/
