# Supplementary material for: Gene Cascade Shift and Pathway Enrichment in Rat Kidney Induced by Acarbose Through Comparative Analysis
Source: Front Bioeng Biotechnol. 2021 May 21;9:659700. doi: 10.3389/fbioe.2021.659700 (PMC8176958; doi:10.3389/fbioe.2021.659700)

**Supplementary Figure 1.** Principal Components Analysis (PCA) of acarbose and water samples. PCA transforms observations of potential correlated variables into values of linearly uncorrelated variables through orthogonal transformation, and plots the two principle components responsible for the most data variability. The first two principal components showed that the total variance of the dataset GSE59913 was 61.8%.


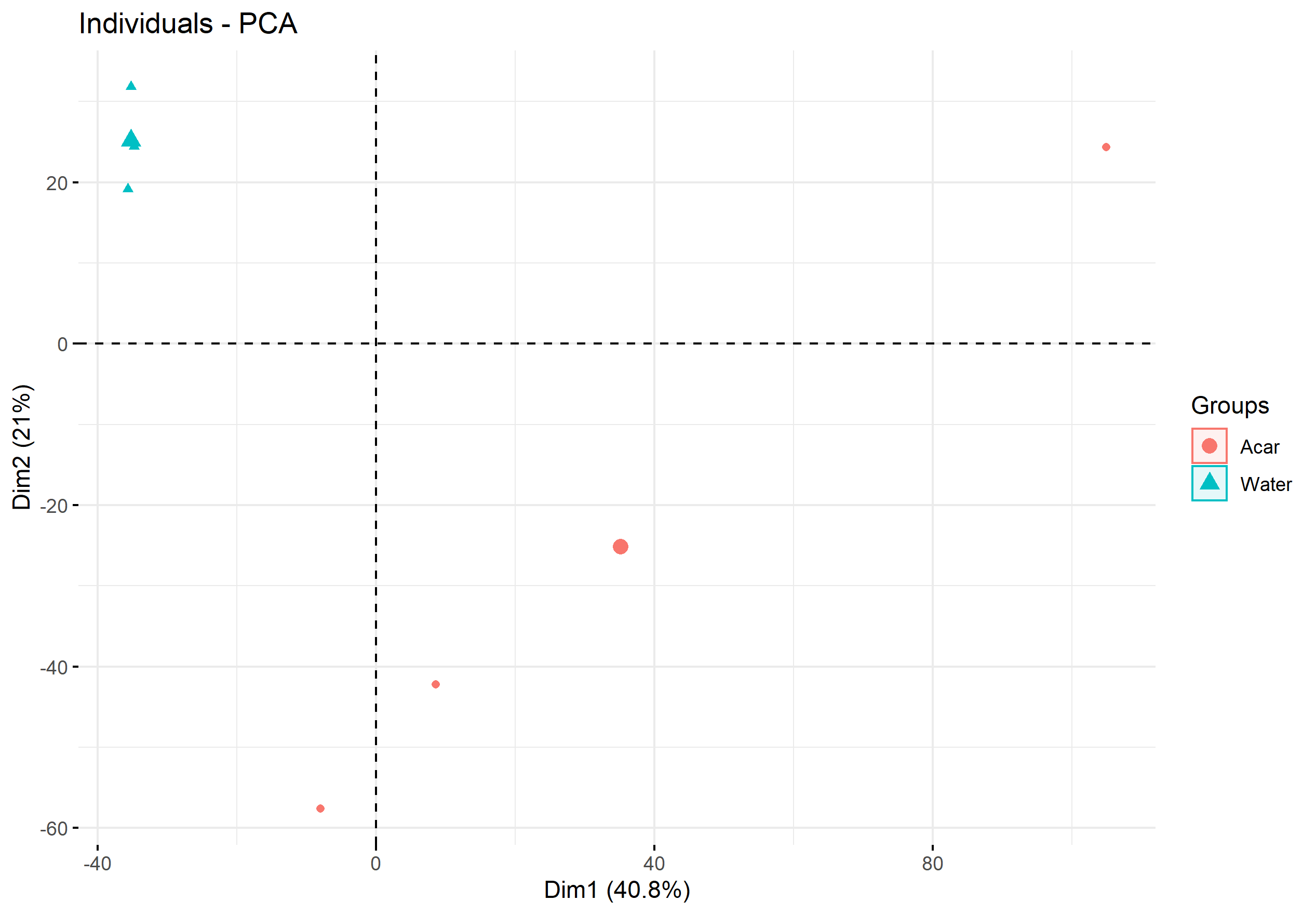

Supplement: Supplementary file 1 [file Data_Sheet_1.DOCX]
