# Supplementary material for: Gene Cascade Shift and Pathway Enrichment in Rat Kidney Induced by Acarbose Through Comparative Analysis
Source: Front Bioeng Biotechnol. 2021 May 21;9:659700. doi: 10.3389/fbioe.2021.659700 (PMC8176958; doi:10.3389/fbioe.2021.659700)
Supplement: Supplementary file 2 [file Data_Sheet_2.pdf]

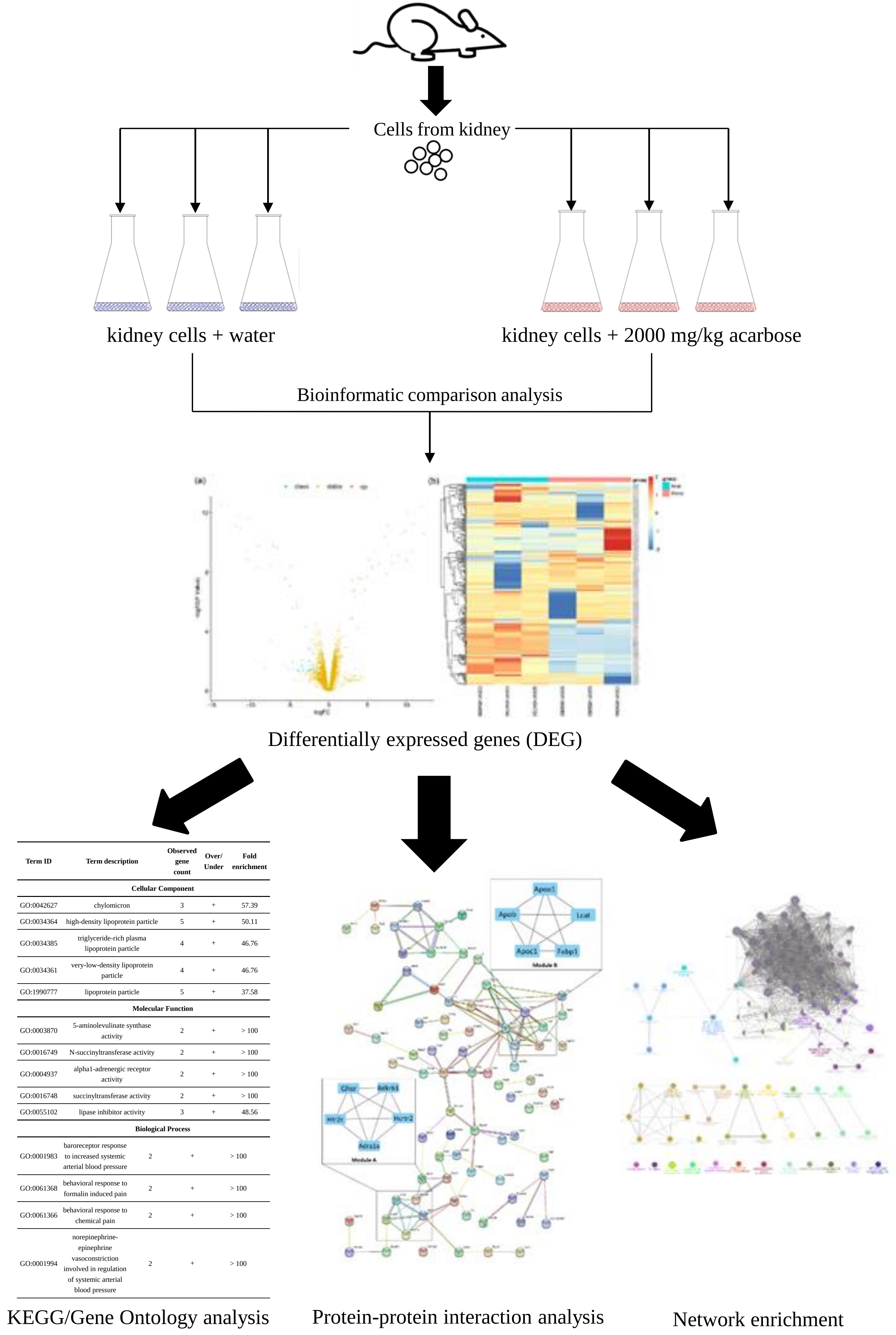

Term ID	Term description	Observed gene count	Over/ Under	Fold enrichment
Cellular Component				
GO:0042627	chylomicron	3	+	57.39
GO:0034364	high-density lipoprotein particle	5	+	50.11
GO:0034385	triglyceride-rich plasma lipoprotein particle	4	+	46.76
GO:0034361	very-low-density lipoprotein particle	4	+	46.76
GO:1990777	lipoprotein particle	5	+	37.58
Molecular Function				
GO:0003870	5-aminolevulinate synthase activity	2	+	> 100
GO:0016749	N-succinyltransferase activity	2	+	> 100
GO:0004937	alpha1-adrenergic receptor activity	2	+	> 100
GO:0016748	succinyltransferase activity	2	+	> 100
GO:0055102	lipase inhibitor activity	3	+	48.56
Biological Process				
GO:0001983	baroreceptor response to increased systemic arterial blood pressure	2	+	> 100
GO:0061368	behavioral response to formalin induced pain	2	+	> 100
GO:0061366	behavioral response to chemical pain	2	+	> 100
GO:0001994	norepinephrine-epinephrine vasoconstriction involved in regulation of systemic arterial blood pressure	2	+	> 100
