# Supplementary material for: Gene Cascade Shift and Pathway Enrichment in Rat Kidney Induced by Acarbose Through Comparative Analysis
Source: Front Bioeng Biotechnol. 2021 May 21;9:659700. doi: 10.3389/fbioe.2021.659700 (PMC8176958; doi:10.3389/fbioe.2021.659700)
Supplement: Supplementary file 3 [file Table_1.DOCX]

**Supplementary Table 1.** Neuroactive ligand-receptor interaction pathway is the most significantly enriched KEGG pathway.

| **Category** | **KEGG_PATHWAY** |
| --- | --- |
| Term | rno04080: Neuroactive ligand-receptor interaction |
| Count | 9 |
| % | 9.89 |
| P Value | 9.61×10^-4^ |
| Genes | GRM5, HCRTR2, SSTR5, ADRB3, P2RX3, ADRA1A, GLP2R, GHSR, HTR2C |
| List Total | 56 |
| Pop Hits | 292 |
| Pop Total | 7749 |
| Fold Enrichment | 1.12 |
| Bonferroni | 0.12 |
