# Supplementary material for: Gene Cascade Shift and Pathway Enrichment in Rat Kidney Induced by Acarbose Through Comparative Analysis
Source: Front Bioeng Biotechnol. 2021 May 21;9:659700. doi: 10.3389/fbioe.2021.659700 (PMC8176958; doi:10.3389/fbioe.2021.659700)
Supplement: Supplementary file 4 [file Table_2.DOCX]

**Supplementary Table 2.** DEGs analyzed by CytoHubba to determine key genes responsible for the gene expression cascade alteration. 12 algorithms were utilized for identification of the most reliable hub genes, and 46 hub genes were selected totally. MCC: Maximal Clique Centrality; DMNC: Density of Maximum Neighborhood Component; MNC: Maximum Neighborhood Component; EPC: Edge Percolated Component. More than one method was utilized for identifying hub since the biological network is heterogeneous.

| MCC | DMNC | MNC | Degree | EPC | BottleNeck | EcCentricity | Closeness | Radiality | Betweenness | Stress | Clustering-  Coefficient |
| --- | --- | --- | --- | --- | --- | --- | --- | --- | --- | --- | --- |
| *Apoc1* | *Apoc1* | *Apoa1* | *Apob* | *Apob* | *Apob* | *Plk1* | *Apob* | *Apob* | *Apob* | *Apob* | *Ccni* |
| *Lcat* | *Lcat* | *Apob* | *Apoa1* | *Apoa5* | *Plk1* | *Cdk1* | *Cdk1* | *Plk1* | *Hspa4* | *Hp* | *Tk1* |
| *Ghsr* | *Ghsr* | *Fabp1* | *Cdk1* | *Apoa1* | *Hspa4* | *Apob* | *Plk1* | *Cdk1* | *Hp* | *Hspa4* | *Nr0b2* |
| *Bdkrb1* | *Bdkrb1* | *Apoa5* | *Fabp1* | *Fabp1* | *Hp* | *Hspa4* | *Apoa1* | *Hspa4* | *Alas2* | *Alas2* | *Apol9a* |
| *Hcrtr2* | *Hcrtr2* | *Hp* | *Hp* | *Apoc1* | *Cdk1* | *Psma3* | *Hp* | *Psma3* | *Plk1* | *Plk1* | *A2m* |
| *Ugt2b17* | *Ugt2b17* | *Apoc1* | *Htr2c* | *Hp* | *Psma3* | *Akap7* | *Hspa4* | *Ccni* | *Cdk1* | *Alox15* | *Alas1* |
| *Plk1* | *Plk1* | *Cyp1a2* | *Apoa5* | *Cdk1* | *Alas2* | *Cenpt* | *Fabp1* | *Tk1* | *Alox15* | *Cdk1* | *Dao* |
| *Cdk1* | *Cdk1* | *Cyp3a18* | *Hspa4* | *Plk1* | *Fkbp5* | *Ccni* | *Apoc1* | *Hp* | *Fkbp5* | *Fkbp5* | *Ghsr* |
| *Hsd3b* | *Hsd3b* | *Htr2c* | *Apoc1* | *Lcat* | *Slc6a4* | *Ensa* | *Apoa5* | *Apoa1* | *Slc6a4* | *Slc6a4* | *ste2* |
| *Ccni* | *Ccni* | *Adra1a* | *Cyp1a2* | *Afm* | *Alox15* | *Tk1* | *Psma3* | *Fabp1* | *Psma3* | *Psma3* | *Ugt2b17* |
